# Element-tracing of mineral matters in *Dendrobium officinale* using ICP-MS and multivariate analysis

**DOI:** 10.1186/s40064-016-2618-2

**Published:** 2016-07-04

**Authors:** Nannan Zhu, Shen Han, Chunning Yang, Jixu Qu, Zhirong Sun, Wenjie Liu, Xiaomin Zhang

**Affiliations:** School of Chinese Materia Medica, Beijing University of Chinese Medicine, No. 6 South Road, Beijing, 100102 People’s Republic of China; Beijing Entry-Exit Inspection and Quarantine Bureau, Beijing, 100026 People’s Republic of China

**Keywords:** *Dendrobium officinale*, Mineral elements, Principal component analysis, Cluster analysis, ICP-MS

## Abstract

Rare studies have been performed to trace the mineral elements in *Dendrobium officinale*. In this study, we aim to trace the mineral elements in *D. officinale* collected from ten geographical locations in China. ICP-MS system was used for simultaneous determination of mineral elements. Principal component analysis was performed using the obtained data in the quantification of mineral contents. Cluster analysis was performed using the Ward’s method. Several of essential microelments were detected in *D. officinale*, including ferrum (Fe), manganese (Mn), zinc (Zn), chromium (Cr), nickel (Ni) and vanadium (V). Among these elements, three elements (i.e. Fe, Mn and Zn) were highly and simultaneously detected in the *D. officinale* collected from the ten locations. The level of Ni was positively associated with that of Zn (r = 0.986, *P* < 0.01). The level of titanium (Ti) was positively associated with that of V (r = 0.669, *P* < 0.05), and negatively associated with Cr (r = −0.710, *P* < 0.05). In addition, the level of Mn was positively associated with that of barium (r = 0.749, *P* < 0.05). Further, the level of Fe was positively associated with that of Ni (r = 0.664, *P* < 0.05), Zn (r = 0.742, *P* < 0.05), and rare earth elements (r = 0.847, *P* < 0.01), respectively. Three eigenvalues explained about 86.60 % of the total variance, which contributed significantly to the explanation of cumulative variance. Cluster analysis indicated the cultivars were categorized into 3 clusters. Ni, Zn, Fe, Cr, Ti and rare earth elements were designated as the characteristic elements. Cultivars collected from Yulin, Menghai, and Shaoguan ranked the top 3 in the comprehensive scores, indicating the content of the mineral elements was comparatively higher in these locations.

## Background

*Dendrobium officinale*, one of the perennial epiphytic herbs, has been commonly used as an ingredient for the nutrition product in China with an aim to enhance the immune system and body strength (Guo et al. 2013). In the past decades, severe shortage of natural *D. officinale* has been noticed with the strict environmental condition demanded by *D. officinale* and the excessive collection. Recently, extensive effort has been made on the artificial cultivation of *D. officinale* in several provinces in China, including Zhejiang, Guangdong, Yunan and Guangxi. However, the quality of *D. officinale* has been reported to be affected by the geographical conditions, especially the type and content of mineral elements which have been considered to be closely associated with the pharmaceutical features of *D. officinale* (Ding et al. 2009). Nevertheless, rare studies have been performed to trace the mineral elements in *D. officinale*.

Element-tracing is very important in the formation or preparation of active chemical constituents present in food and nutrition product (Yamashita et al. 2005; Maiga et al. 2005). The chemical constituents are partially associated with the medicinal and nutritional properties of the herbs (Nookabkaew et al. 2006). In this study, elements-tracing was performed in *D. officinale* cultivars collected from ten different locations in China. The potential correlation between the traced elements was investigated, and principal component analysis (PCA) was performed to evaluate the correlation matrix. Further, clustering analysis was performed to investigate the geographical features of the cultivars. Our study could contribute to the quality control of main components in *D. officinale*.

## Methods

### Materials

*Dendrobium officinale* cultivars were collected in August 2013 from ten locations in China, including Xingyi (Guizhou Province), Shaoguang (Guangdong Province), Yulin (Guangxi Province), Xinanjiang (Zhejiang Province), Menghai (Yunnan Province), Simao (Yunnan Province), Honghe (Yunnan Province), Yuxi (Yunnan Province), Wenshan (Yunnan Province), and Dehong (Yunnan Province). At least three independent but parallel cultivars were collected from each geographical location. The identification of the cultivars was performed by Professor ZR Sun.

### Sample preparation

Sample preparation was performed as previously described by Tokalıoğlu (2012). Briefly, the cultivars were washed with tap water thoroughly, followed by distilled water. At least three cultivars obtained from each location were used for the sample preparation. After drying at 105 °C, the *D. officinale* was grounded into powder using a mortar and sieved using a 60-grid sifter. Then the powder (0.20 g) was added to a conical flask containing 10 mL nitric acid and perchloric acid mixture (nitric acid/perchloric acid = 5:1). Subsequently, the mixture was heated to 150 °C using a hot plate until complete mineralized. Afterwards, the temperature was modulated to 200 °C. Upon complete digestion, the flask was obtained and washed using demineralized water. Finally, the mixture was transferred to a 10 mL color comparison tube for further analysis.

### Determination of mineral contents using ICP-MS

An Agilent 7500 ICP-MS system was used for simultaneous determination of iron (Fe), manganese (Mn), zinc (Zn), titanium (Ti), chromium (Cr), nickel (Ni), vanadium (V), barium (Ba), and rare earth elements (REEs), including scandium (Sc), yttrium (Y), lanthanum (La), cerium (Ce), praseodymium (Pr), neodymium (Nd), samarium (Sm), europium (Eu), gadolinium (Gd), terbium (Tb), dysprosium (Dy), holmium (Ho), erbium (Er), thulium (Tm), ytterbium (Yb), and lutetium (Lu). The voltage for the ion lens was set at 6 V. The gas flow rate in the spray chamber was 0.88 L min^−1^. The power output for the RF generator was 1100 W. The auxiliary gas flow rate was 1.2 L min^−1^. The nebulizer gas flow rate of the plasma was 16 L min^−1^. The certified reference materials for calibration used were GBW3041 (Ti), GBW(E)080216 (V), GBW(E)080257 (Cr), GBW(E)080157 (Mn), GBW08616 (Fe), GBW08618 (Ni), GBW08620 (Zn), GBW(E)080243 (Ba), as well as GSB-04-1750-2004 (Sc) and GSB-04-1789-2004 (for rare earth elements except Sc). All the certified reference materials (in solution) were purchased from the National Institute of Metrology (Beijing, China). Blank control (n = 3) were carried out in the same way. GBW10052 Tea Certified Reference Material was used to validate the accuracy of the method. All the procedures were performed in triplicate.

### Statistical analysis

Data were presented as mean ± standard deviation. PCA was performed using the obtained data in the quantification of mineral contents. SPSS 19.0 software was used for the data analysis. *P* < 0.05 indicated statistical difference.

## Results

### Concentration of elements in *D. officinale*

The accuracy of the method was tested by analyzing of certified reference material (GBW10052). Table 1 summarized the concentration of mineral elements in *D. officinale* cultivars. Several of essential microelments were detected in *D. officinale*, including Fe, Mn, Zn, Cr, Ni and V. Among these elements, three elements (i.e. Fe, Mn and Zn) were highly and simultaneously detected in the *D. officinale* collected from the ten locations. The highest content of Fe, Mn and Zn was determined in the cultivars collected from Yulin (Fe: 779 ± 24 μg g^−1^), Shaoguan (Mn: 884 ± 23 μg g^−1^) and Yulin (Zn: 366 ± 24 μg g^−1^), respectively.

**Table 1:** Mineral contents in the *D. officinale* collected from 10 geographical locations in China

Location	Ti (μg g^−1^)	V (μg g^−1^)	Cr (μg g^−1^)	Mn (μg g^−1^)	Fe (μg g^−1^)	Ni (μg g^−1^)	Zn (μg g^−1^)	Ba (μg g^−1^)	REEs (μg g^−1^)
Xingyi	11.07 ± 2.23	7.45 ± 1.27	2.38 ± 1.02	102.89 ± 15.79	356.28±	2.54 ± 0.29	97.69 ± 15.52	7.94 ± 1.25	0.54 ± 0.14
Shaoguan	14.37 ± 3.25	9.87 ± 2.35	2.36 ± 0.98	884.95 ± 23.36	273.98 ± 12.87	4.72 ± 0.45	330.89 ± 25.98	30.87 ± 6.54	0.25 ± 0.04
Yulin	14.87 ± 3.45	12.37 ± 2.48	2.85 ± 0.87	82.48 ± 9.34	779.56 ± 23.56	4.59 ± 0.88	365.85 ± 23.96	10.65 ± 2.12	0.56 ± 0.14
Xinanjiang	7.30 ± 1.23	6.67 ± 1.56	1.89 ± 0.28	117.96 ± 10.69	225.32 ± 15.29	2.65 ± 0.57	125.01 ± 12.56	18.68 ± 2.54	0.25 ± 0.09
Menghai	17.06 ± 3.56	11.17 ± 2.56	2.45 ± 0.56	492.01 ± 14.78	265.89 ± 20.65	4.69 ± 0.59	288.75 ± 25.12	18.62 ± 4.98	0.37 ± 0.08
Simao	12.25 ± 2.89	6.78 ± 2.06	3.37 ± 0.98	455.47 ± 23.25	115.87 ± 23.69	1.05 ± 0.32	26.58 ± 5.21	8.56 ± 1.25	0.23 ± 0.08
Honghe	13.10 ± 3.23	10.56 ± 2.25	3.43 ± 0.96	178.02 ± 12.58	137.89 ± 19.36	0.76 ± 0.12	7.39 ± 2.14	10.63 ± 1.28	0.25 ± 0.06
Yuxi	12.57 ± 3.69	6.45 ± 1.45	3.21 ± 0.87	275.74 ± 13.47	99.59 ± 9.87	0.87 ± 0.21	27.27 ± 9.07	10.87 ± 2.14	0.13 ± 0.02
Wenshan	11.25 ± 2.25	5.37 ± 1.45	3.23 ± 0.75	437.25 ± 25.12	98.89 ± 13.65	0.84 ± 0.24	27.80 ± 6.98	17.25 ± 2.36	0.23 ± 0.06
Dehong	12.09 ± 3.26	11.12 ± 2.24	3.58 ± 0.76	30.35 ± 8.96	138.98 ± 13.67	0.61 ± 0.14	5.08 ± 0.97	13.51 ± 3.12	0.17 ± 0.05

The concentration is a sum of elements. REE included: scandium, yttrium, lanthanum, cerium, praseodymium, neodymium, samarium, europium, gadolinium, terbium, dysprosium, holmium, erbium, thulium, ytterbium and lutetium

*REE* rare earth element

The content of REEs was also detected in the plants, and the content of REE was less than 1 μg g^−1^ (Table 1). The cultivars collected from Xingyi showed the highest REE content, while the lowest REE was revealed in the cultivars collected from Simao.

### Correlation between the content of mineral elements

**Fig. 1:**
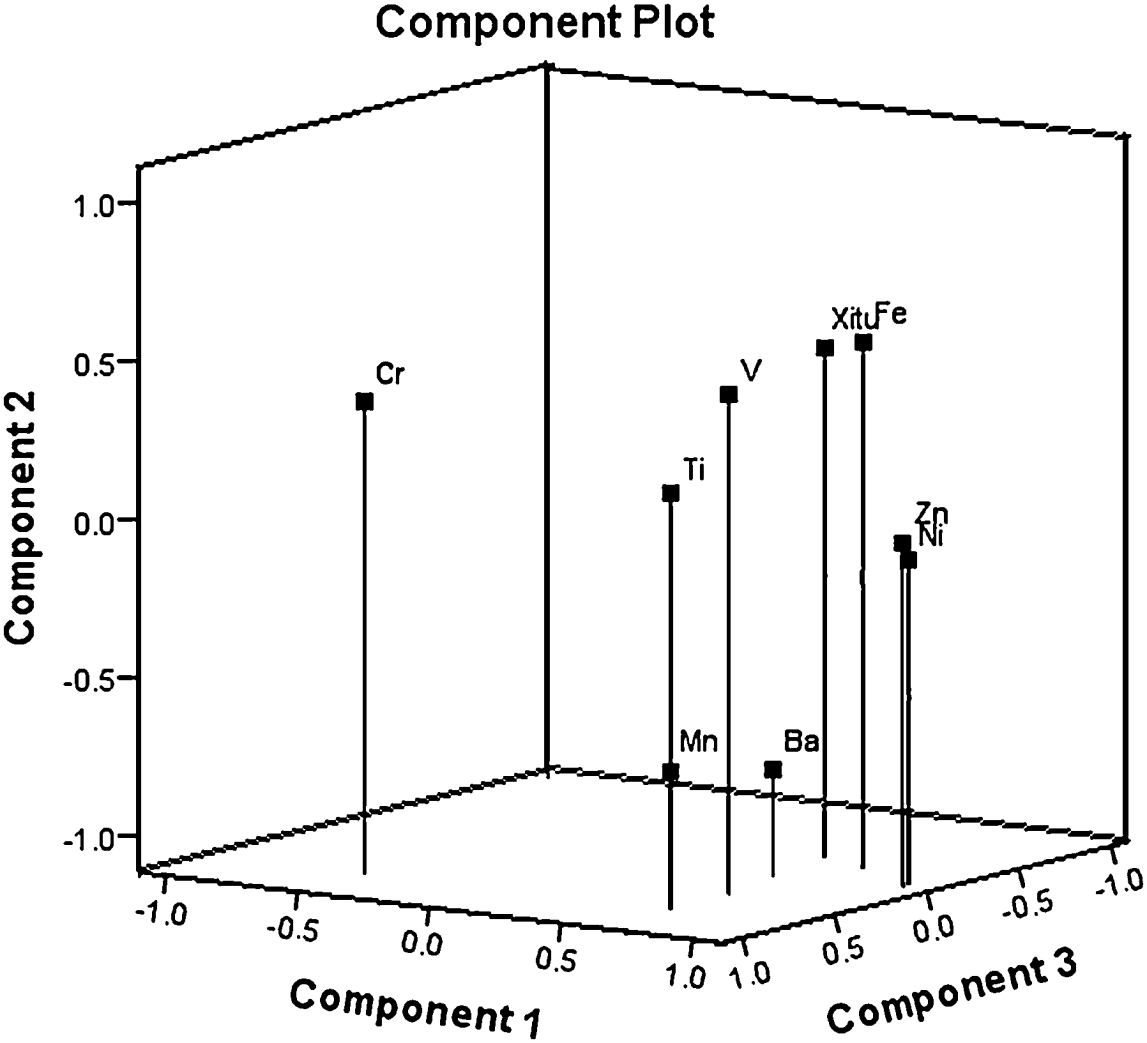
Results of the principal component analysis of *D. officinale*. Three eigenvalues (component 1–3) explained about 86.60 % of the total variance

In this study, we evaluated the metal to metal correlation. As revealed in Fig. 1, the level of Ni was positively associated with that of Zn (r = 0.986, *P* < 0.01). The level of Ti was positively associated with that of V (r = 0.669, *P* < 0.05), and negatively associated with Cr (r = −0.710, *P* < 0.05). In addition, the level of Mn was positively associated with that of Ba (r = 0.749, *P* < 0.05). Further, the level of Fe was positively associated with that of Ni (r = 0.664, *P* < 0.05), Zn (r = 0.742, *P* < 0.05), and REEs (r = 0.847, *P* < 0.01), respectively.

### PCA of mineral elements

PCA was performed to evaluate the correlation matrix using SPSS 19.0 software. The data set was autoscaled. The results indicated that three eigenvalues (PC1-3) explained about 86.60 % of the total variance, which contributed significantly to the explanation of cumulative variance. Therefore, the first three eigenvalues were selected for further analysis. The first factor has high loadings for Ni and Zn, and explained about 46.28 % of the variance. The second factor has high loadings for Fe and REEs, and explained about 69.86 % of the variance. Meanwhile, the third factor has high loadings for Cr and Ti, and explained about 86.60 % of the variance (Table 2). A three-dimensional plot of the PC loading was displayed in Fig. 1, and the relationship among the heavy metals could be observed clearly.

**Table 2:** Principal component analysis of mineral elements in *D. officinale*

Item	Principal component analysis		
	1	2	3
Zscore (Ti)	0.630	0.200	0.702
Zscore (V)	0.624	0.464	0.378
Zscore (Cr)	−0.506	0.380	0.740
Zscore (Mn)	0.404	−0.751	0.380
Zscore (Fe)	0.699	0.546	−0.245
Zscore (Ni)	0.980	−0.090	−0.088
Zscore (Zn)	0.982	−0.031	−0.054
Zscore (Ba)	0.549	−0.779	0.031
Zscore (REE)	0.494	0.495	−0.330
λ	4.165	2.122	1.507
Proportion (%)	46.281	23.582	16.741
Cumulative proportion (%)	46.281	69.863	86.604

### Analysis of major eigenvalues and the rank

The calculation of eigenvalues was performed according to the following formula:

$$\begin{aligned} {\text{F}}_{1} & = 0.31{\text{ZX}}_{1} + 0.31{\text{ZX}}_{2} - 0.25{\text{ZX}}_{3} + 0.20{\text{ZX}}_{4} + 0.34{\text{ZX}}_{5} + 0.48{\text{ZX}}_{6} + 0.48{\text{ZX}}_{7} + 0.27{\text{ZX}}_{8} + 0.24{\text{ZX}}_{9} ; \\ {\text{F}}_{2} & = 0.14{\text{ZX}}_{1} + 0.32{\text{ZX}}_{2} + 0.26{\text{ZX}}_{3} - 0.52{\text{ZX}}_{4} + 0.37{\text{ZX}}_{5} - 0.06{\text{ZX}}_{6} - 0.02{\text{ZX}}_{7} - 0.53{\text{ZX}}_{8} + 0.34{\text{ZX}}_{9} ; \\ {\text{F}}3 & = 0.57{\text{ZX}}_{1} + 0.31{\text{ZX}}_{2} + 0.60{\text{ZX}}_{3} + 0.31{\text{ZX}}_{4} - 0.20{\text{ZX}}_{5} - 0.07{\text{ZX}}_{6} - 0.04{\text{ZX}}_{7} + 0.03{\text{ZX}}_{8} - 0.27{\text{ZX}}_{9} . \\ \end{aligned}$$

**Fig. 2:**
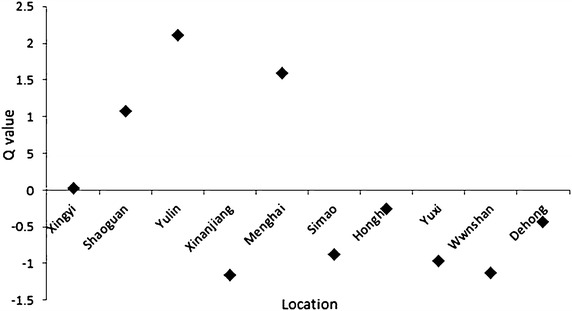
Q values of the cultivars in different locations

According to these formula, a comprehensive expression was obtained as follows:

$${\text{Q}} = \, \left( {\uplambda_{1} \times {\text{F}}_{1} +\uplambda_{2} \times {\text{F}}_{2} +\uplambda_{3} \times {\text{F}}_{3} } \right) \times \left( {\uplambda_{1} +\uplambda_{2} +\uplambda_{3} } \right) - 1,$$

whether Q is the comprehensive score, and λ_1_, λ_2_ and λ_3_ stand for the characteristic root of the major eigenvalues. Cultivars collected from Yulin, Menghai, and Shaoguan ranked the top 3 in the comprehensive scores, indicating the content of the mineral elements was comparatively higher in these locations (Fig. 2).

### Cluster analysis

**Fig. 3:**
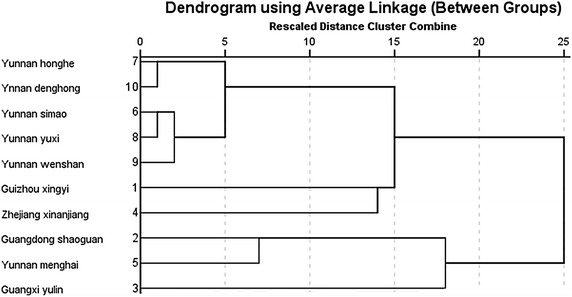
Cluster analysis of mineral elements in *D. officinale*. Cluster analysis was performed using the Ward’s method, and the cultivars were mainly classified into three clusters. Similarities on the quantification of mineral elements were noticed in each cluster

In our study, cluster analysis was performed using the Ward’s method, which revealed the cultivars were mainly classified into three clusters: (1) cultivars collected from Simao, Yuxi, Wenshan, Honghe, and Dehong; (2) cultivars collected from Xingyi and Xinanjiang; and (3) cultivars collected from Shaoguan, Yulin and Menghai. In each cluster, the cultivars showed similarities on the quantification of mineral elements, demonstrating the quantification of *D. officinale* was associated with geographic locations (Fig. 3).

## Discussions

Increasing evidence reveals that certain elements of *D. officinale*, a Chinese herbal plant, could enhance the immune functions and inhibit the cell apoptosis (Xiang et al. 2013; Liu et al. 2011). Recently, *D. officinale* has been commonly applied in the research and development of healthcare products in China, and the content of the mineral elements and microelements is crucial for the quality control of the commercial products. In this study, we traced the elements in the cultivars in different geographical locations.

Extensive studies indicates that mineral elements and microelements are crucial for the health and disease prevention (Deng et al. 2009; Grace et al. 1999). For example, metal microelements, including Zn, Cu, Ag and Au, have been reported to involve in the main biochemical parameters such as ATP-ase activity, transmembrane potential and respiratory activity (Rieznichenko et al. 2008). Additionally, low zinc in diet was associated with decrease in resting metabolic rate (Wada and King 1986). Further, iron played important roles in the regulation of body temperature and energy production in order to response to the reduced environmental temperature (White-Ziegler et al. 2007).

In the present study, several mineral elements (i.e. Fe, Mn, Zn, Cr, Ni, V, Ti, Ba and REEs) were simultaneously detected in the *D. officinale* collected from 10 geographical locations in China using ICP-MS. According to the quantification analysis, the content of the mineral elements varied in the *D. officinale* collected from different locations. Three elements (i.e. Fe, Mn, and Zn) were simultaneously detected with high content in these cultivars. For the biological properties of these elements, four elements (i.e. Fe, Mn, Zn, and Ni) have been reported with anti-tumor activity (Bernhardt et al. 2009). Fe has been considered to be essential for the hematopoiesis in human bodies (Li et al. 2011; Morikawa et al. 1995). In addition, Zn and Mn are closely associated with the immune function in vivo (Huskisson et al. 2007; Gajula et al. 2011). Further, Cr is crucial for the maintenance of blood sugar and lipid metabolism (Dogukan et al. 2009). In this study, we also evaluated the correlation among the mineral elements that were simultaneously detected. The results revealed that the quantification of Ni was positively correlated with that of Zn. Meanwhile, a positive correlation was noticed in Ti and V, Mn and Ba, Fe and Ni, as well as Fe and Zn, respectively.

PCA, describing the relationship of multi-dimensional data between variables and objects, has been commonly used for the evaluation of distributing features of mineral elements and microelements (Ringner 2008; Joliffe and Morgan 1992). In this study, three eigenvalues explained about 86.60 % of the total variance were obtained, among which Ni and Zn were highly loaded in the first eigenvalue, Fe and REE were highly loaded in the second eigenvalue, and Ti and Cr were highly loaded in the third eigenvalue, respectively. Therefore, we concluded that these elements could be considered as the representative elements of *D. officinale*, and the top 3 cultivars with the most content of mineral elements were those obtained from Yulin, Menghai, and Shaoguan.

As revealed by clustering analysis, the cultivars collected from ten geographical locations were categorized into three clusters. *D. officinale* collected from Simao, Yuxi, Wenshan, Honghe, and Dehong was categorized into the same cluster due to close locations, and the content of mineral elements was similar. *D. officinale* collected from Xingyi and Xinanjiang was categorized into the same cluster as the cultivars were grown under a tropical monsoon climate and a montanic environment. *D. officinale* collected from Shaoguan, Yulin and Menghai was categorized into the same cluster as these cultivars were distributed at the same latitude. Interestingly, the cultivars collected from Yunan province were not categorized into the same cluster. This indicated a genetic variation may present in the *D. officinale* cultivars of various geographical locations.

## Conclusions

Nine mineral elements were simultaneously detected in the *D. officinale* collected from ten geographical locations in China using ICP-MS, which were categorized into three eigenvalues using the PCA, and elements (e.g. Ni, Zn, Fe, REE, Cr, and Ti) were designated as the characteristic elements. Cluster analysis indicated the cultivars collected from ten locations were categorized into three clusters, which demonstrated that geographical location was closely associated with the variance of the cultivars.
